# Treatment needs and acknowledgement of illness – importance for satisfaction with psychiatric inpatient treatment

**DOI:** 10.1186/1472-6963-8-103

**Published:** 2008-05-14

**Authors:** Knut W Soergaard, Mary Nivison, Vidje Hansen, Terje Oeiesvold

**Affiliations:** 1 Nordland Hospital Trust, Bodo/Institute of Clinical Medicine, Dept. of Clinical Psychiatry, University of Tromso, Norway; 2 University Hospital of Northern-Norway, Tromso, Norway; 3 Institute of Clinical Medicine, Dept. of Clinical Psychiatry, University of Tromso, Norway; 4 Nordland Hospital Trust, Bodo/Institute of Clinical Medicine, Dept. of Clinical Psychiatry, University of Tromso, Norway

## Abstract

**Background:**

Patient satisfaction is an important, but controversial part of health service evaluation. This study dealt with how acknowledgement of illness and treatment needs effected the distribution of positive, neutral and negative evaluations in a group of first time admitted patients to a psychiatric hospital.

**Method:**

The participants filled out a standardized user satisfaction form before discharge. The number of positive, neutral and negative evaluations for each participant was calculated and used as dependent variables in analyses (Classification Tree) where acknowledgement of illness (The Patients' Experience of Hospitalisation Questionnaire) and treatment needs (HoNOS) were used as explanatory variables in addition to a number of potential confounders.

**Results:**

Different constellations of variables explained the three dependent variables. The number of positive scores was a function of age and worry (PEH); neutral scores were explained by HoNOS rated social needs and GAF (functional scale), both at admission. Outcome (GAF functional scale) and age explained the number of negative scores.

**Conclusion:**

(1) Moderately high negative correlations between positive and neutral scores, and between positive and negative scores, together with a positive correlation between the number of negative and neutral ratings was interpreted to mean that neutral scores sometimes function as undercommunicated negative evaluations. These could better be studied by qualitative methods. (2) The worry subscale (PEH) was important in identifying the majority of patients with the highest numbers of positive scores (patients older than 27.5 yrs with high worry score at admission.). The most dissatisfied group was characterised by denial of both mental problems and need for treatment. (3) Patients with high scores on the HoNOS Social subscale had the highest number of neutral scores. To the extent that neutral evaluations have negative connotations, treatment should focus more effectively on the patients' social needs. (4) The smallest number of negative scores was found among older patients with high functional improvement (GAF F). (5) Increasing age consistently predicted higher satisfaction. A better understanding of why younger patients are more dissatisfied is needed.

## Background

Measurement of patient satisfaction is criticised for giving an optimistic and somewhat limited view of the services, but is increasingly considered to be a necessary supplement to administrative and clinical measures of healthcare quality. Concerns are raised both regarding the concept of patient satisfaction and the methods used to measure it. Satisfaction is commonly used as an outcome measure, but may be even more important as an intervening variable that influences therapeutic usefulness, help-seeking, compliance and service utilisation. In addition to revealing characteristics of the services, satisfaction is closely related to characteristics of the users. In studies of satisfaction these are generally restricted to demography, often supplemented by diagnosis and outcome in routine evaluations. There are no definite conclusions about how demography and diagnosis influence treatment satisfaction [1-3]. Some older studies showed that psychosis, drug abuse or suicidal behaviour [4,5]and chronic problems [6] were associated with less satisfaction. Satisfaction also seems to be related to outcome [7-9] and to self-reported improvement [1,10], especially if measured at the same time and with comparable methods [10]. Treatment satisfaction may be affected by subjective factors such as expectation about the treatment, self-esteem, attribution, insight and how one feels about life in general. Ignoring such factors may be acceptable in routine evaluations but are detrimental to the development of a deeper understanding of user satisfaction, and may contribute to the contradictory conclusions that characterise research into patients' views about their care. Subjective quality of life [11] and how one feels about life in general seem to be associated with satisfaction [12]. Conflicting results have been found for expectations about the treatment [1,2,13]. The importance of insight for the understanding of satisfaction is not fully explored [14].

The aim of this study was to explore how some rarely used variables – Acknowledgement of Illness and Patient Needs – were associated with satisfaction in a group of first-time admitted patients. Acknowledgement of illness is a complex phenomenon closely related to insight and refers to what happens when a person and/or his/her significant others interpret mental and social signals related to stress and dysfunction in efforts to give meaning to suffering and to find solutions to problems. Acknowledgement may result in help-seeking, enhanced compliance with treatment, better treatment outcomes and may lead to higher satisfaction. In this study acknowledge of illness was measured using the Patients's Experience of Hospitalization Questionnaire [15]. Patients' needs are relevant because how the services deal with the needs of different groups of patients may disclose qualities of the treatment systems. Treatment needs are usually studied from a diagnostic point of view, but when needs are defined as an individual's requirements for being able to live according to demographically relevant cultural standards, psychiatric diagnoses have some shortcomings. Psychiatric inpatients usually have a variety of different needs (often grouped as clinical, cognitive, behavioural and social), and inpatient treatment may deal better with some of them (e.g. symptoms) than with others (e.g. activities, housing). Associations between unmet needs and satisfaction with treatment were for example found in a group of discharged long-term patients [16]. In the present study, treatment needs were defined and measured using Health of the Nation Outcome Scales (HoNOS) [17].

It has long been recognised that user satisfaction is multidimensional and that patients may be more satisfied with certain aspects of the treatment and not with others. However, we did not focus on how the patients evaluated specific aspects of the services (staff, medication, information etc), but on factors that were associated with cumulative numbers of positive, neutral, negative and extreme ratings of satisfaction with inpatient treatment. The main goals of the study were to explore how acknowledgement of illness and patient needs were related to these four conceptualisations of satisfaction. Theoretically, acknowledgement of illness, defined as subjective awareness of need for treatment, need for hospitalisation, worrying and hope, may be associated with higher satisfaction owing to for example better compliance and placebo-effects, but also with more negative evaluations due to disappointment and lack of recovery. With regard to needs, we expected that inpatient treatment would be better tailored to patients with symptom needs than for example Social and Behavioural needs, and that symptom recovery would be related to a higher number of positive scores.

## Method

### Participants

The study was a prospective investigation of all first-time admitted patients to the psychiatric departments of Nordland Hospital in a one year period and a 12-month follow-up period. The hospital is located in Northern Norway and serves a population of 239 000 inhabitants. All first-time admitted patients between ages of 18 and 65 were included (N = 295). Criteria for inclusion were: no previous admissions to the admitting hospital and signed informed consent to participate. Exclusion criteria were: discharged less than 3 days after admission, lack of language competency, and cognitive impairment such as dementia, serious mental retardation or other mental incapacity preventing the individual from giving an informed written consent. The main causes for nonparticipation was lack of language competence (immigrants, refugees etc), cognitive and clinical problems that made interviews impossible and – especially – short length of stay (0–3 days). The rationale for excluding patients with short stays was that lengthy in- and out-interviews and a number of self-administrated questionnaires within a framework of 0 to 3 days were unfeasible. This gave 202 eligible participants. Of these 55 refused to participate and 30 of the consenting patients did not return the satisfaction questionnaire. 117 patients participated in the study (58%). Compared with non-participators, the participants had significantly shorter stays (26.4 vs 41.3 says, p = .006), but this difference disappeared when the comparison was restricted to patients with stays longer than 3 days. There were no differences with respect to age, sex, clinical diagnosis and portions of committed to non committed, married to non-married and employed to benefit recipients. The study was approved by the Regional Ethical Committee in Norway.

### Measures

Patient satisfaction was measured at discharge with the Norwegian version of the SPRI form. The SPRI is a 50-item self-rating instrument developed by the Swedish Institute for Development of Health Services [18]. It has been extensively used in studies of patient satisfaction in the Nordic countries. In addition to demographic and descriptive information (previous admissions, legal status etc.), it covers staff-patient relations, information and influence on own treatment, treatment regimens and the treatment program as a whole. The response scales have 5 operationally defined steps from very poor (1) to very good (5).

Some items also include a 0-score signifying no treatment/not relevant. In this study we did not want to focus on domain-specific satisfaction (e.g. satisfaction with ward staff, treatment as a whole etc.), but on the number of negative ratings (1 and 2, 0 when relevant – e.g. no information), the number of neutral ratings (3), the number of positive ratings (4 and 5). 0-scores were sometimes interpreted as negative evaluations, for example when the patients reported that they had not received information about the treatment, that their viewpoints about the treatment had not been listened to, that they had not been told about their rights to complain, had not been allocated a key worker etc. On other items 0-scores meant no treatment/not relevant, for example when the patients had not had contact with a social worker, a psychologist or received group therapy. We also constructed a variable identifying two groups of patients: (i) extreme positives (patients with scores two standard deviations above the average number of positive ratings) and (ii) extreme negatives (two standard deviations below the average number of negative ratings) and used this variable as dependent in multivariate analyses. The purpose was to find which variables distinguished the two groups. Some of the forms filled out by the patients had missing values which in turn affected the number of forms used in the different analyses. Acknowledgement of illness was measured soon after admission with the Patient's Experience of Hospitalisation Questionnaire (PEH) [15]). The PEH is an 18 items pencil and paper measure that assesses the patient's perceived need for treatment and hospitalisation, extent of worry about illness, as well as illness-related issues. In addition to the total score (PEH Total) the questionnaire consists of four subscales: Need for Treatment, Need for Hospitalisation, Worry, and Hope. Items are scored from 1 ("Strongly agree") to 4 ("Strongly disagree"). A number of items are reversed to reduce response bias. A Norwegian translation of the Health of the Nation Outcome Scale (HoNOS) [17] was used to measure treatment needs. The HoNOS consists of 12 items and is scored from 1 to 4 giving a maximum possible score of 48 points. A high score indicates greater needs. HoNOS has 4 sub-scales: Behaviour (aggression/disruptive behaviour, self harm, substance use), Impairment (cognition, physical health), Symptoms (hallucinations and delusions, depression and other symptoms) and Social function (social relations, general functioning, housing, activities). It can be used to characterise the clinical and social needs of the patients [19]. All patients were diagnosed using the Mini-International Neuropsychiatric Interview (M.I.N.I.-PLUS.)[20], Norwegian version 5.0.0 [21]. M.I.N.I. was developed in Europe and U.S.A. as a short diagnostic instrument for generating DSM-IV criteria diagnoses convertible to ICD-10 diagnosis. The M.I.N.I.-Plus is an extended version of M.I.N.I. that includes information on specific phobias and has an expanded psychosis module. The M.I.N.I.-Plus is built up of 15 modules corresponding to diagnostic categories and collects information along 23 axis-I problem areas in relation to current and past symptoms. An experienced psychologist (not employed at the hospital) set the diagnoses on the basis of M.I.N.I-Plus interviews done by the interviewers. Symptom and function level at admission and at discharge were measured with the Global Assessment of Functioning (symptom and functioning scale – GAF f and GAF s) [22]. The GAF is often considered to be of dubious value in clinical practice, but a recent study [23] found that although GAF used in routine clinical settings was unreliable, used by trained raters in research situations reliability is good.

### Procedure

After admission the ward staff and admitting doctor assessed all first-time patients and determined if their condition allowed inclusion in the study. Eligible participants were given written and oral information about the study and asked to respond within the next 24 hours. After giving written consent, interviews were planned within as short a time span as possible. The self-rating scales were distributed after conclusion of the interview. A shorter interview was performed at discharge. Five experienced clinical staff members (3 psychiatric nurses, a psychologist and a psychiatrist) without therapeutic relations to the patients did the interviews. Before the study they underwent systematic training in the use of the measures included in the study. Consecutive reliability checks were performed.

### Statistical methods

Non-parametric statistics (Mann-Whitney, Kruskal-Wallis, Spearman correlations) were used in the univariate analyses. The classification tree (CT) [24] was chosen for the multivariate analyses due to a marked deviation from many of the parametric requirements of most of the variables (ordinal/nonlinearity, normality, colinearity, distribution of residuals etc). CT is used to predict membership of cases in classes or groups. The predictors are examined recursively to find the one that gives the best classification by splitting the sample into subgroups (nodes). The basic structure consists of a root node that is divided into smaller and smaller branches or groups until the stopping criteria are met (the terminal nodes). The CRT procedure used here divides the data into segments that are as homogenous as possible with respect to the dependent variable. In contrast to discriminant analyses that treats all independent variables simultaneously, the CT performs the univariate splits by examining the effects of predictors one at a time. CT can be computed for categorical predictors, continuous predictors or any combination of the two types. When there are many potential explanatory variables, classification trees can give a clear picture of the structure of the data and interaction among the variables. The method is widely used in applied sciences such as medicine and psychology. Interpretation is simplified by graphic presentations. The numbers of positive, neutral and negative scores in addition to the two extreme groups were used as dependent variables. We used differences in GAF- and HoNOS ratings from admission to discharge as measures of change. Potential confounders used in the multivariate analyses were gender, age, length of education, marital status, type of housing, length of stay at the hospital, type of admission (involuntary vs. voluntary), and diagnosis. The GAF and HoNOS ratings were determined by the interviewers. Data analyses were performed with SPSS 15.00.

## Results

### Univariate analyses

The correlation between the number of positive and neutral satisfaction scores on the SPRI was -.66 (p < .01), between positive and negative scores -.68 (p < .01), and between neutral and negative scores .49 (p < .01). There was a tendency for female patients to have more positive scores (16.1 vs. 14.5, NS), fewer neutral (5.7 vs. 4.3. p = .047) and negative scores (4.3 vs 3.4 NS) than male patients. The correlation between age and number of positive scores was .44 (p < .01), neutral scores -.30 (p < .01) and negative scores -.27 (p < .01). None of the PEH scales (total and subscales) correlated significantly with the number of positive, neutral and negative evaluations and extreme scores on the SPRI. Significant correlations between the dependent variables and the HoNOS scales were as follows: The number of positive scores correlated .20 with Impairment (p = .012) and -.23 with Behavioural needs (p = .012). Neutral scores correlated -.21 with Impairment (p = .026). The number of negative scores correlated .20 with Behavioural needs (p = .032). There were no significant correlations between any of the ratings of satisfaction and either GAF F admission or GAF S admission. GAF S improvement correlated .23 (p. = .025) with the number of positive scores. GAF F improvement correlated .25 (p. = .018) with the number of positive scores, -.21 (p. = .041) with the number of neutral scores, and -.27 (p = .011) with the number of negative scores. There were no diagnoses-related differences on any of the dependent variables.

### Multivariate analyses

Figure 1 shows the results of the classification tree (CT) procedures on the number of positive scores. The average number of positive scores was 15.2. Age was the primary explanatory variable with patients older than 27.5 having an average of 16.9 positive scores. Low score on the PEH variable Worry indicates high worrying, and the group with the highest number of positive ratings (22.1) was a small group of high worrying patients between 27.5 and 48.5 yrs. Patients younger than 27.5 yrs had an average of 10.7 such scores.

The average number of neutral scores was 5.0 (figure 2). The highest number of neutral scores (8.6) was found in a small group of patients with an average of 5.5 such needs. Few Social needs and low functional score (GAF F) at admission was associated with a small number of such scores (4.0). Figure 3 shows the analysis of the number of negative ratings. The average number of such scores was 3.9. The most important variable was functional improvement during the stay (GAF F). The group with most negative ratings had negative GAF F improvement ratings which indicated a deterioration in functioning during their stays. Functional improvement interacted with age in identifying the group with the fewest negative scores: Patients with high functional improvement and age above 48.5 yrs (mean score was 1.8).

Age was the primary variable determining if a patient scored in an extreme category on the SPRI. In the most positive group 8 of 14 patients were older than 44.5 yrs. In the most dissatisfied group all patients (N = 13) were younger than/equal to 44.5 yrs.

## Discussion

The primary aim of the study was to investigate how some rarely used variables were related to four different operationalisations of patient satisfaction. The variables were acknowledgement of illness (PEH) and needs (HoNOS). Four subscores from each of these were used in the analyses in addition to the total scores. By restricting the sample to first-time admitted patients, we controlled for ratings based on previous hospital experiences. A number of potential confounders were taken into consideration.

The variables of primary interest in the present study – acknowledgement of illness (PEH) and needs (HoNOS) appeared as predictors behind the number of positive and neutral scores, respectively. The worry dimension of the PEH (worrying about present conditions such as illness, losing friends, and being unable to work) was important in identifying the majority of patients with the highest numbers of positive scores: patients older than 27.5 yrs with high worry score at admission and who, due to that, probably felt a strong need for treatment. The HoNOS Social subscale identified patients with the highest number of neutral scores. Social needs refer to problems relating to other people, activities of daily life, dwelling and occupation/activities. If – as we suggest below – neutral scores often function as underexpressed negative evaluations, a possible explanation is that such needs are difficult to alleviate due to different organisational context of hospitals and municipality services.

Different constellations of explanatory variables were found behind the dependent variables. This confirm the multidimensionality of the concept of patient satisfaction. The number of positive scores was a function of two variables: age and worry (PEH). The basic trend was that the number of positive ratings increased with increasing age. Contrasting subgroups were made up of younger age patients (below 27.5 yrs) with an average of 10.7 positive scores and older patients (above 48.5 yrs) who at admission were worried about their mental status, and who in average had 22.1 such ratings. A different constellation of variables explained the main distribution of neutral scores. The main variable was Social needs (HoNOS). A high score here indicates problems relating to other people, with activities of daily life, dwelling and occupation/activities. Patients with many social needs had an average of 8.6 such scores, whereas patients with few social needs and low functional GAF score at admission had the lowest number of neutral ratings. Functional improvement during the stay (GAF F) was the main variable associated with the number of negative scores. Patients with improvement ratings lower or equal to minus 9 points – that is a deterioration compared with the admission score – had the highest number of negative scores. High age interacted with improvement so that the lowest number of negative scores was found for older patients (above 48.5 yrs) with high (above 9 points) improvement scores. Age was the most important variable determining if a patient scored in an extreme category on the SPRI. In the most positive group 57% of the patients were older than 44.5 yrs, whereas in the most dissatisfied group all patients were younger than/equal to 44.5 yrs.

Age appeared in three of the analyses: twice in the number of positive scores, once in the number of negative scores and as the only variable in the Extreme group analyses. Similar results have been reported by [7,9,25,26,25,26] although not consistently [27]. Hypothetically, the influence of age may result from factors related both to the age of the patients (older patients may adapt more easily to the relatively inflexible ward routines, be more compliant towards the treatment, be more respectful etc. whereas younger patients may be more defiant, less accepting of their situation) and to differences in age between younger patients and the staff (the average age of the staff in this study was 38.6 yrs).

We found clinical outcome to be the primary variable behind negative ratings: Negative functional improvement – that means deterioration – was associated with many negative evaluations (8.4). The lowest number of such scores (1.8) was found among older patients (above 49.4 yrs) with high functional improvement score. Outcome was absent in the other analyses (positive, neutral and extreme). When we compared the group with the highest number of negative evaluations negative evaluations (N = 8) with the rest of the sample, the first group had significantly higher scores on the PEH-scales (except hope) whereas the differences on the clinical scales (HoNOS and GAF) were trifling. The PEH scores suggest that these highly dissatisfied patients did not acknowledge mental problems and need for treatment. Because it was restricted to the number of negative scores, our results do not directly confirm studies that show good clinical outcome to be associated with higher satisfaction [13,9]. Due to shared method variance associations between two or more constructs such associations may occur when the measurements of outcome and satisfaction are performed at the same time and with similar methods [28]. Our study used outcome ratings by the interviewers whereas satisfaction was assessed by the patients themselves, and thus was not affected by this problem.

It is intriguing why GAF-rated clinical outcome was restricted to the number negative and not to positive evaluations. The mean number of negative ratings in the patients who were most dissatisfied was 2.4 times that of the rest of the patients. These patients were homogenously extreme in two other ways: in addition to denying their mental problems and need for treatment, they also underwent the strongest negative clinical change between admission and discharge. This made them very dissimilar to more motivated patients who generally, but in varying degrees, experienced good clinical outcome, and who expressed this in positive scores on most of the measured aspects.

Of a total of 34 different SPRI-items, the average number of positive, neutral and negative satisfaction scores was 15.2, 5.0 and 3.0, respectively (the difference between the maximum and the factual total scores was mainly explained by items scored as "not relevant" when the patient for example had not received special types of treatment or services). The high number of positive ratings confirms the results from many similar studies [27,29]. It is rare that more than 10% of the service recipients give scores in the dissatisfied range [5,30]. The positive predominance has caused concerns about the validity of satisfaction measurements and about the influence of desirability bias, ingratiation response sets and other psychological mechanisms [31], and is an argument for also using more open ended methods [25,32]. And related to this: We found significant negative correlations between the number of positive and the number of negative (-.60) and neutral (-.59) ratings, and a positive correlation between negative and neutral ratings (.40). Positive scores also correlated positively with GAF symptom and functional improvement, whereas there were negative correlations between good GAF-rated functional outcome and the number of negative and neutral evaluations. The association between the neutral and negative ratings, their negative associations to the number of positive scores, and their correlations with functional improvement, suggests that neutral evaluations may carry negative connotations and sometimes function as undercommunicated [25] negative appraisals. Berghofer [11] noticed that many of their outpatients frequently used neutral ratings and suggested that psychiatric patients may have difficulty in making critical comments. A Swedish study [32] compared questionnaire and interview ratings of satisfaction in the same group of patients and found that the patients when interviewed expressed more negative evaluations than they did in the questionnaires. It is possible that patients in interviews are better able to articulate diffuse or vaguely negative experiences than when filling out questionnaires.

### Methodological weaknesses

The attrition rate was high and may have led to non-response biases. The use of language competence as an exclusion criterion leaves out an important group of patients who represent special challenges to the treatment and the hospital routines. The patients' evaluation of their treatment may have been altered by their mental problems. However, evaluations by the patients are (not without exceptions [28]), considered to be good indicators of the quality of the services [33]. Although anonymous, the results may also have been biased due to acquaintance with staff members. Neutral evaluations may hide a critical attitude that is best studied by qualitative methods.

## Conclusion

(1) There were moderately high [34] negative correlations between the number of positive and neutral satisfaction scores, and between positive and negative scores. A positive correlation was found between the number of negative and neutral ratings. We interpreted this to mean that neutral scores may sometimes function as undercommunicated negative evaluations. Qualitative methods should be used to identify negative experiences among patients.

(2) The worry subscale of the PEH was important in identifying the majority of patients with the highest numbers of positive scores: patients older than 27.5 yrs with high worry score at admission. The most negative group in the study (most negative ratings) were characterised by denial of both mental problems and need for treatment.

(3) The HoNOS Social subscale was the primary variable behind the number of neutral scores: patients with many such scores had the highest number of neutral scores. To the extent that neutral evaluations have negative connotations, treatment should focus more effectively on the patients' social needs.

(4) Age appeared in three of the analyses: twice in the number of positive scores, once in the number of negative scores and as the only variable in the Extreme group analyses. Increasing age consistently predicted higher satisfaction. A better understanding of why younger patients are more dissatisfied is needed.

(5) Clinical outcome (GAF F) was the primary variable behind negative ratings: Small functional improvement – or rather deterioration – predicted many negative evaluations. The lowest number of negative scores was found among older patients with high functional improvement (GAF F) score. Outcome was absent in the other analyses (positive, neutral and extreme groups).

(6) In contrast to some other studies diagnosis did not influence the satisfaction scores.

## Competing interests

The authors declare that they have no competing interests.

## Authors' contributions

KWS participated in the design of the study, interviewed patients, performed the statistics and wrote the manuscript. MN participated in the design of the study, translated the PEH, and helped to draft the manuscript. VH participated in the design of the study, helped to draft the manuscript. TO participated in the design of the study, helped to draft the manuscript. All authors read and approved the final manuscript.

## Pre-publication history

The pre-publication history for this paper can be accessed here:


